# Marine Macroalgal Diversity Assessment of Saba Bank, Netherlands Antilles

**DOI:** 10.1371/journal.pone.0010677

**Published:** 2010-05-21

**Authors:** Mark M. Littler, Diane S. Littler, Barrett L. Brooks

**Affiliations:** Department of Botany, Smithsonian Institution, Washington, D. C., United States of America; University of California San Diego, United States of America

## Abstract

**Background:**

Located in the Dutch Windward Islands, Saba Bank is a flat-topped seamount (20–45 m deep in the shallower regions). The primary goals of the survey were to improve knowledge of biodiversity for one of the world's most significant, but little-known, seamounts and to increase basic data and analyses to promote the development of an improved management plan.

**Methodology/Principal Findings:**

Our team of three divers used scuba to collect algal samples to depths of 50 m at 17 dive sites. Over 360 macrophyte specimens (12 putative new species) were collected, more than 1,000 photographs were taken in truly exceptional habitats, and three astonishing new seaweed community types were discovered. These included: (1) “Field of Greens” (N 17°30.620′, W 63°27.707′) dominated by green seaweeds as well as some filamentous reds, (2) “Brown Town” (N 17°28.027′, W 63°14.944′) dominated by large brown algae, and (3) “Seaweed City” (N 17°26.485′, W 63°16.850′) with a diversity of spectacular fleshy red algae.

**Conclusions/Significance:**

Dives to 30 m in the more two-dimensional interior habitats revealed particularly robust specimens of algae typical of shallower seagrass beds, but here in the total absence of any seagrasses (seagrasses generally do not grow below 20 m). Our preliminary estimate of the number of total seaweed species on Saba Bank ranges from a minimum of 150 to 200. Few filamentous and thin sheet forms indicative of stressed or physically disturbed environments were observed. A more precise number still awaits further microscopic and molecular examinations in the laboratory. The expedition, while intensive, has only scratched the surface of this unique submerged seamount/atoll.

## Introduction

**Figure 1:**
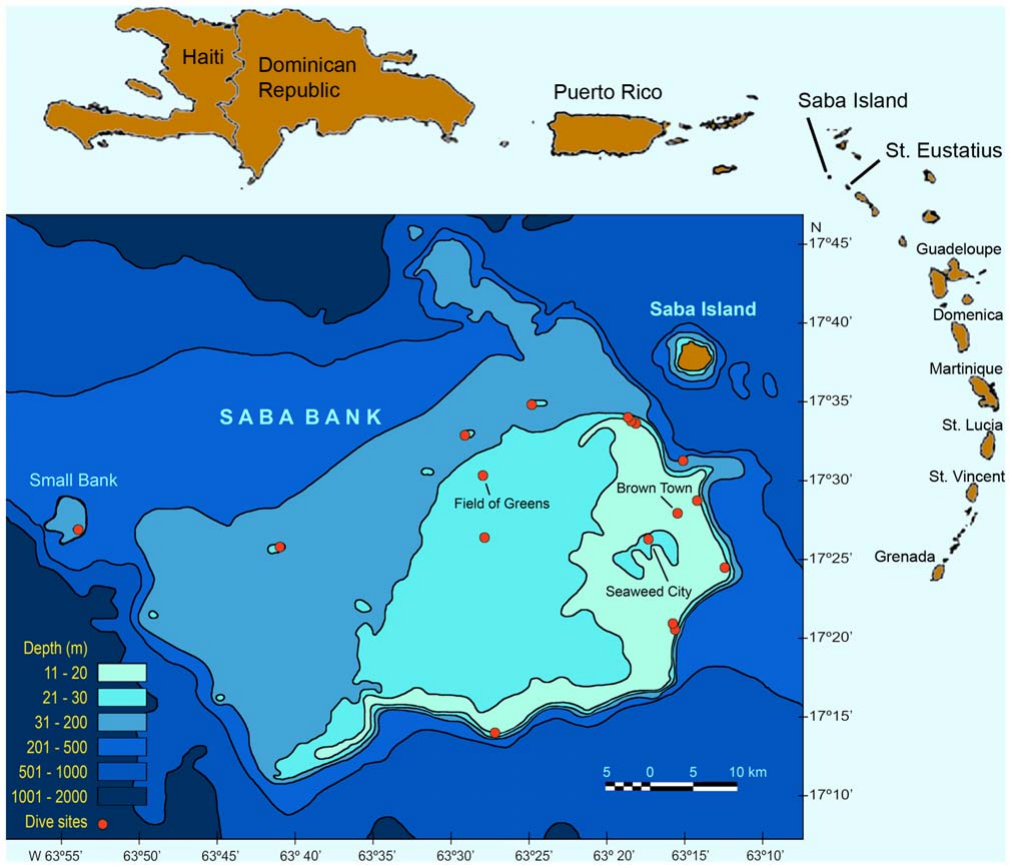
Saba Bank. Bathymetric chart of Saba Bank in relation to Caribbean islands, showing the 17 collecting locations with the unique algal sites labeled as Field of Greens, Brown Town and Seaweed City.

Located in the Dutch Windward Islands about 250 km SE of Puerto Rico, Saba Bank is a flat-topped seamount rising 1.8 km from the surrounding sea floor (Figure 1). The dimensions of the Bank are extremely large for an atoll/seamount; the platform above the 200 m isobath is 65 km in length by 40 km in breadth, and it covers an area of 2,200 km^2^. A large portion of the Bank (about 225 km^2^ in area) lies between 12 and 20 m in depth and contains a rich assemblage of biota in varied habitats; most of the remainder of the summit is between 20 and 50 m in depth and remains virtually unexplored. Saba Bank meets the true definition of a seamount being isolated by deep water; in this case the nearest islands are Saba and St. Eustatius. Except for the fact that it does not break the water surface, Saba Bank is a classic atoll consisting of a submerged mountain crowned at the summit with a ring of actively growing coral reefs. Saba Bank is the largest atoll in the Atlantic Ocean Basin and one of the three largest atolls on earth [1].

Because of its distance from large land masses, Saba Bank is relatively free of the problems that are degrading many Caribbean reef systems, mainly sedimentary run off from poor farming practices or rapid construction, increased nutrient loading from an ever-increasing human population, and overfishing by local fisherman. There is little room for agriculture on Saba Island's five square miles; the ∼1,500 residents rely mainly on income from tourists that come to scuba dive. The fishing community also generates a significant income from fishing nearby Saba Bank.

However, Saba Bank is threatened by the oil trans-shipment depot on nearby St. Eustatius Island. Supertankers stop there to transfer oil to tanks used to fill smaller ships that are able to enter countries without deep-water ports. Rather than pay mooring fees at St. Eustatius, tankers drop anchor on Saba Bank at no monetary cost (but often with detrimental biological damage). The immediate concerns for the Bank includes anchor damage, prop blast, and abrasion by large oil tankers maneuvering off the petroleum trans-shipment facilities on St. Eustatius, petroleum spillage and subsequent use of dispersants, possible overfishing for certain species, and exploration for petroleum reserves (so far unsuccessful). At the same time, the Bank's fisheries and dive operations are economically significant to the small community on Saba Island (about 1,500 residents) that has direct responsibility for its management.

Although it ranks among the major topographic structures of the Caribbean, Saba Bank is one of the most poorly known. A series of interpretations of its structure and origin [2]-[4] has culminated in the view that the Bank essentially duplicates the atolls of the Pacific [5]. Small samples of fishes and possibly other organisms have been collected by passing research vessels (the R/V Oregon in 1968 and 1969 and the R/V Pillsbury in 1969), and Macintyre and others carried out a preliminary survey in 1970 [6]. A Dutch expedition in 1974 utilized echo soundings, bottom sampling, and scuba to characterize the reef communities [5]. The hydrographic research vessel HNMNS Tydeman carried out a survey of the seafloor in 1996, which resulted in the current nautical charts. More recently in a post-hurricane rapid assessment, three sites on Saba Bank's eastern edge showed [7] average macroalgal biomass to be inversely related to that of herbivorous fishes., with turf algae and crustose coralline algae relatively more abundant (8 and 11 times more, respectively).

Saba Bank was the focus of a joint program initiated by the Department of Environment and Nature of the Netherlands Antilles, Conservation International, and the Protocol for Specially Protected Areas and Wildlife (SPAW) of the Convention for the protection and development of the marine environment in the Wider Caribbean (Cartagena Convention), of the Caribbean Regional Seas Program under the United Nations Environmental Program (UNEP). The primary goals of the survey were to: improve knowledge of biodiversity for one of the world's most significant coral/algal-capped seamounts; increase basic information in support of improved management; provide data and analyses to support the development of a marine zoning plan; and contribute to a petition to the International Maritime Organization to designate appropriate parts of Saba Bank as Particularly Sensitive Areas.

Much of the paucity of data stems from difficulties in logistics and hazardous sea conditions characteristic of Saba Banks. On some days, the small marine park patrol boats and fishing boat struggled more than 56 km to designated preselected dive sites in 4-m seas.

## Results

**Figure 2:**
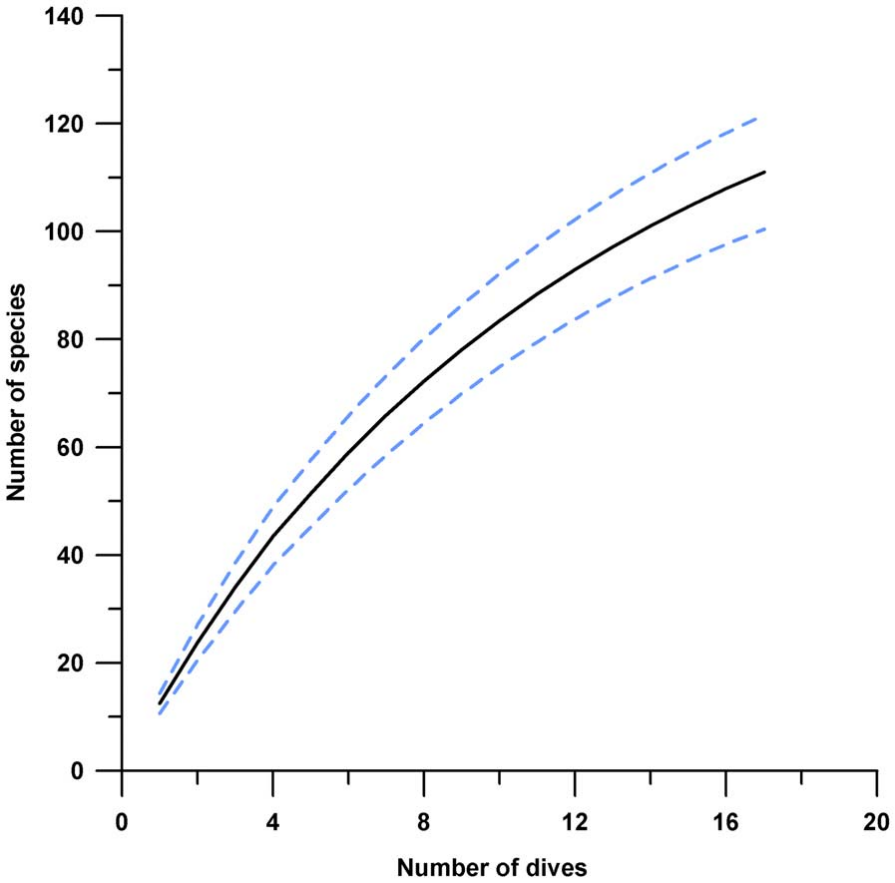
Species accrual curve for algae on Saba Bank. Several undetermined species (spp.) not included in the check list were used in this graph. Sample based rarefaction curve shown in black is based on 17 dive sites. The Mau Tao estimator [8] of expected richness with 95% confidence intervals shown in blue. The curve reaches no asymptote, indicating a likelihood that many additional algal species remain to be found.

Three hundred sixty specimens of macrophytes were collected by our team. Many of these include numerous other archived but uncounted species of epiphytes and smaller microscopic taxa. Our preliminary estimate of the number of macrophyte species collected from Saba Bank ranges upward from 150 to 200. A more precise number awaits long hours of microscopic and molecular examinations in the laboratory. However, the present annotated checklist of 98 taxa (Table 1) includes 43 Rhodophyta, 26 Chlorophyta, 26 Phaeophyceae, and 3 Cyanophyta (Cyanobacteria). The species accrual curve for these collections (Figure 2) [8] was steadily inclined after the 17 dive sites (Table 2), and showed no clear asymptote, indicating Saba Bank has many more species to be collected. It is important to note that there were few filamentous and thin sheet forms indicative of stressed or physically disturbed environments observed [9], [10].

**Table 1:** Annotated list of algae identified from Saba Bank arranged by major groups.

Bluegreens (Cyanobacteria)	
Species name	Collection #
*Lyngbya polychroa* (Meneghini) Rabenhorst	D&ML 67418
*Phormidium* cf. *dimorphum* Lemmerman	D&ML 67417
*Symploca hydnoides* (Harvey) Kützing	D&ML 67420

Browns (Phaeophyceae)	
Species name	Collection #
*Dictyopteris justii* JV Lamouroux	D&ML 67488
*Dictyota cf. ciliolata* Sonder ex Kützing	D&ML 67439
*Dictyota ciliolata* Sonder ex Kützing	D&ML 67451
*Dictyota crispata* JV Lamouroux	D&ML 67500
*Dictyota guineensis* (Kützing) P & H Crouan	D&ML 67491
*Dictyota hamifera* Setchell	D&ML 67419
*Dictyota humifusa* Hörnig, Schnetter &Coppejans	D&ML 67250
*Dictyota menstrualis* (Hoyt) Schnetter, Hörning & Weber-Peukert	D&ML 67475
*Dictyota mertensii* (Martius) Kützing	D&ML 67452
*Dictyota pinnatifida* Kützing	D&ML 67300
*Lobophora variegata* (JV Lamouroux) Womersley ex Oliveira	D&ML 67241
*Sargassum hystrix* J Agardh	D&ML 67296
*Sargassum platycarpum* Montagne	D&ML 67411
*Sargassum polyceratium*? Montagne	D&ML 67454
*Stypopodium zonale* (JV Lamour.) Papenfuss	D&ML 67414
*Zonaria tournefortii* (JV Lamour.) Montagne	D&ML 67350

Greens (Chlorophyta)	
Species name	Collection #
*Avrainvillea asarifolia* Børgesen	D&ML 67302
*Caulerpa cupressoides* (Vahl) C Agardh	D&ML 67569
*Caulerpa mexicana* Sonder ex Kützing	D&ML 67530
*Caulerpa microphysa* (Weber-van Bosse) J Feldmann	D&ML 67499
*Caulerpa prolifera* (Forsskål) JV Lamour	D&ML 67576
*Caulerpa racemosa* f. *lamourouxii* (Turner) Weber-van Bosse	D&ML 67504
*Caulerpa serrulata* (Forsskål) J Agardh	D&ML 67379
*Caulerpa webbiana* Montagne	D&ML 67387
*Codium taylorii* PC Silva	D&ML 67490
*Dictyosphaeria cavernosa* (Forsskål) Børgesen	D&ML 67492
*Halimeda* cf. *tuna* f. *platydisca* (Decaisne) ES Barton	D&ML 67274
*Halimeda discoidea* Decaisne	D&ML 67460
*Halimeda goreaui* WR Taylor	D&ML 67272
*Neomeris annulata* Dickie	D&ML 67507a
*Neomeris mucosa* MA Howe	D&ML 67507b
*Penicillus capitatus* Lamarck	D&ML 67571
*Penicillus dumetosus* (JV Lamour.) Blainville	D&ML 67529
*Rhipocephalus phoenix* (J Ellis & Solander) Kützing	D&ML 67512
*Udotea cyathiformis*	D&ML 67359
*Udotea dotyi* DS Littler & MM Littler	D&ML 67575
*Udotea occidentalis*? A Gepp & ES Gepp	D&ML 67573
*Udotea spinulosa* MA Howe	D&ML 67574
*Valonia macrophysa* Kützing	D&ML 67509

Reds (Rhodophyta)	
Species name	Collection #
*Amphiroa tribulus* (J Ellis & Solander) JV Lamouroux	D&ML 67422
*Asteromenia peltata* (WR Taylor) Huisman & A Millar	D&ML 67428
*Champia parvula* (C Agardh) Harvey	D&ML 67331
*Champia salicornioides* Harvey	D&ML 67342
*Chondria dasyphylla* (Woodward) C Agardh	D&ML 67503
*Chondrophycus corallopsis* (Montagne) Nam	D&ML 67566
*Chrysymenia dickieana* J Agardh	D&ML 67541
*Chrysymenia enteromorpha* Harvey	D&ML 67338
*Cryptonemia crenulata* (J Agardh) J Agardh	D&ML 67511
*Dasya antillarum* (MA Howe) AJK Millar	D&ML 67548
*Dasya baillouviana* (SG Gmelin) Montagne	D&ML 67567
*Dasya collinsiana* MA Howe	D&ML 67352
*Dasya crouaniana* J Agardh	no number
*Dasya harveyi* Ashmead ex Harvey	D&ML 67542
*Dasya mollis* Harvey	D&ML 67553
*Dasya punicea* (Zanardini) Meneghini ex Zanardini	D&ML 67326
*Dudresnaya bermudensis* Setchell	D&ML 67400
*Eucheuma isiforme* (C Agardh) J Agardh	D&ML 67324
*Galaxaura rugosa* (J Ellis & Solander) JV Lamouroux	D&ML 67423
*Gelidiopsis intricata* (C Agardh) Vickers	D&ML 67510
*Gracilaria cervicornis* (Turner) J Agardh	D&ML 67514
*Gracilaria cylindrica* Börgesen	D&ML 67317
*Griffithsia schousboei* Montagne	D&ML 67534
*Halymenia floresii* (Clemente y Rubio) C Agardh	D&ML 67543
*Hypoglossum hypoglossoides* (Stackhouse) Collins & Hervey	D&ML 67535
*Jania capillacea* Harvey	D&ML 67501
*Laurencia chondrioides* Börgesen	D&ML 67318
*Laurencia intricata* JV Lamouroux	D&ML 67421
*Laurencia lata* MA Howe & WR Taylor	D&ML 67539
*Meristiella schramiAi* (Crouan & Crouan) Cheney & Gabrielson	D&ML 67497
*Neogoniolithon spectabile* (Foslie) Setchell & Mason	D&ML 67405
*Peyssonnelia conchicola* Piccone & Grunow	D&ML 67517
*Porolithon pachydermum* (Foslie) Foslie	no number
*Scinaia halliae* (Setchell) Huisman	D&ML 67537
*Tricleocarpa fragilis* (Linnaeus) Huisman & Townsend	D&ML 67489
*Wrangelia penicillata* (C Agardh) C Agardh	D&ML 67288
*Wrightiella blodgettii* (Harvey) F Schmitz	D&ML 67322

**Table 2:** Listing of site data.

Site#	Date	Site Name	Latitude	Longitude	Depth m
1	4-Jan-06	Poison Bank (Northeast Reef)	17° 28.756N	63° 13.600W	20-27
2	5-Jan-06	Small Bank South	17° 26.827N	63° 54.057W	37-42
3	5-Jan-06	Rhodolith Reef (middle area)	17° 25.832N	63° 40.962W	38
4	6-Jan-06	Redman Bulge 3 (Overall Bank)	17° 24.599N	63° 11.802W	35
5	6-Jan-06	Seaweed City	17° 26.485N	63° 16.850W	24
6	7-Jan-06	Grouper Bank	17° 33.085N	63° 28.847W	36
7	7-Jan-06	Rendezvous Hill	17° 34.873N	63° 24.450W	23
8	8-Jan-06	Butterfly Reef	17° 14.083N	63° 26.996W	22-37
9	9-Jan-06	Moonfish Bank (Fishpot Surprise)	17° 33.774N	63° 17.897W	19
10	10-Jan-06	Moonfish Bank (Lost Anchor)	17° 33.895N	63° 17.965W	25-28
11	10-Jan-06	Moonfish Bank	17° 33.900N	63° 18.221W	19
12	12-Jan-06	Poison Bank	17° 30.758N	63° 13.692W	29
13	12-Jan-06	Brown Town	17° 28.027N	63° 14.944W	20
14	13-Jan-06	Red Flats	17° 26.359N	63° 27.769W	28
15	13-Jan-06	Field of Greens	17° 30.620N	63° 27.707W	30
16	14-Jan-06	Coral Garden	17° 20.756N	63° 15.036W	23–30
17	14-Jan-06	Conch Valley	17° 21.184N	63° 15.121W	17–22

**Figure 3:**
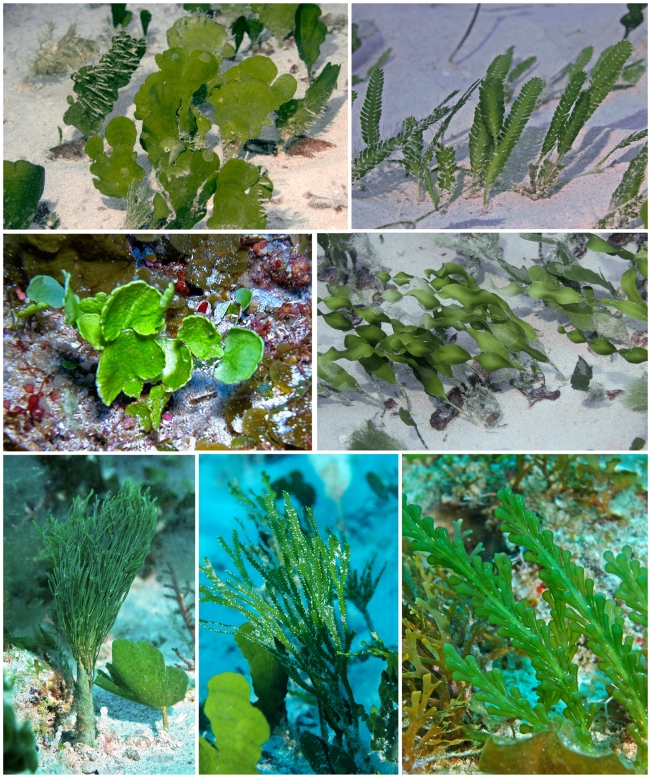
Selected species from “Field of Greens”. A region (> one hectare) of Saba Bank (depth 25 m) that our group named “Field of Greens” because the vast sand plain was dominated by green algae (Chlorophyta). Top left: *Udotea occidentalis*. Top right: *Caulerpa mexicana*. Middle left: *Halimeda* cf. *tuna* forma *platydisca*. Middle right: *Caulerpa prolifera*. Bottom left: *Penicillus dumentosus*. Bottom middle: *Caulerpa cupressoides*. Bottom right: *Caulerpa racemosa* f. *lamourouxii*.

**Figure 4:**
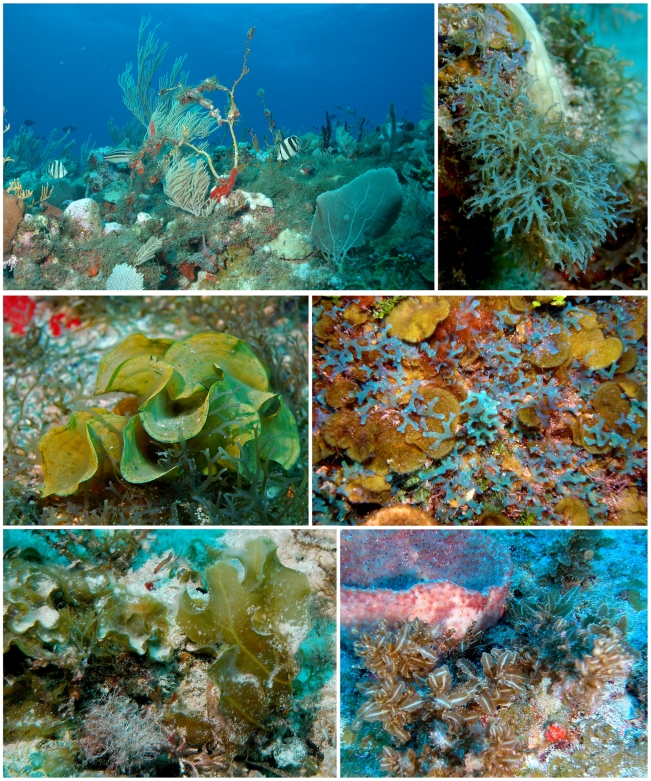
Selected species from “Brown Town”. The large region (> one hectare) of Saba Bank (depth 25 m) nicknamed “Brown Town” because of the domination by brown macroalgae (Phaeophyceae). Top left: Overview of “Brown Town” habitat with over 50% cover of *Dictyota* turf. Top right: *Dictyota hamifera*. Middle left: *Stypopodium zonale*. Middle right: mixture of *Lobophora variegata* and *Dictyota humifusa*. Bottom right: *Dictyopteris justii*. Bottom right: *Sargassum hystrix*.

**Figure 5:**
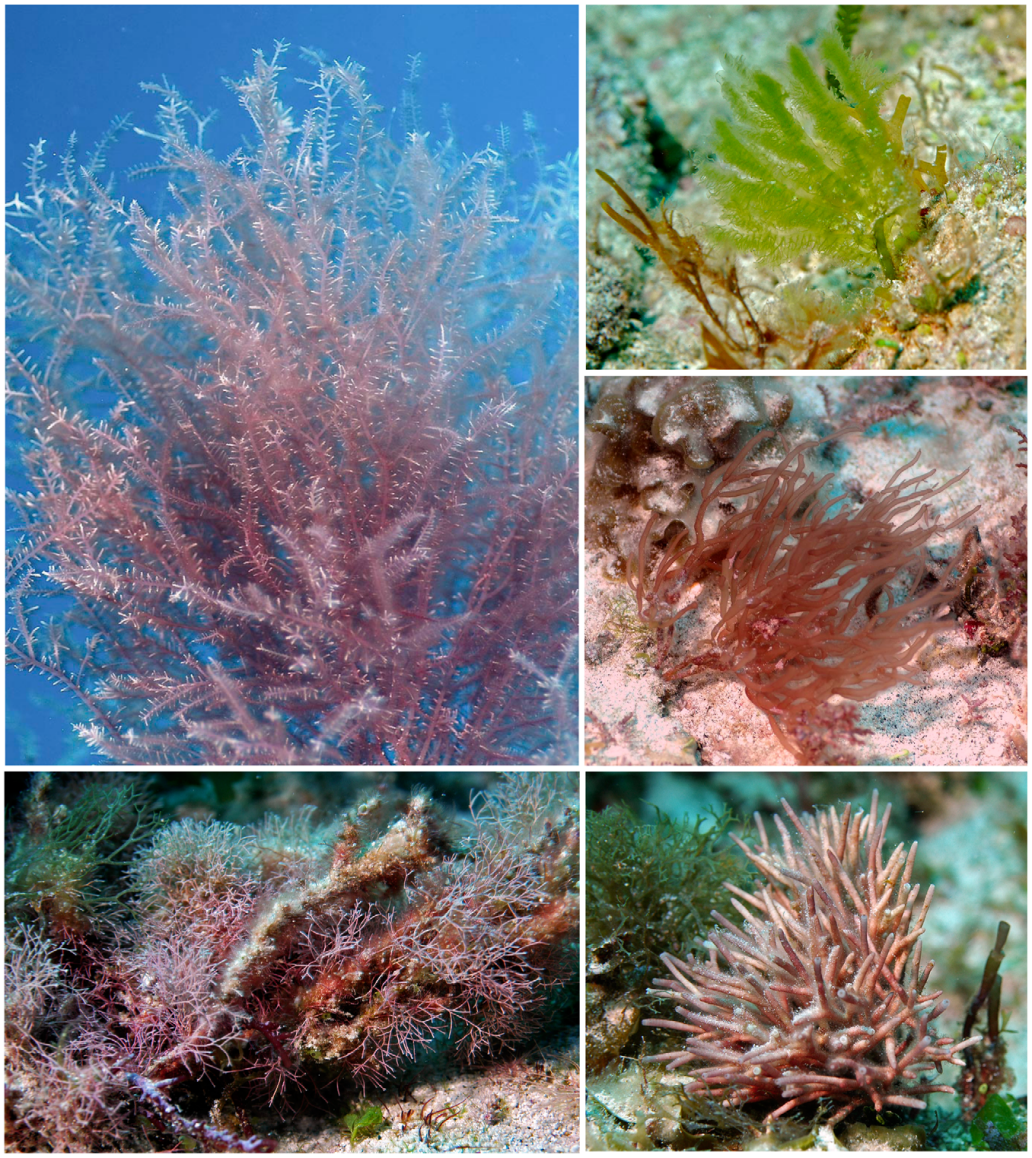
Selected specimens from “Seaweed City”. Specimens dominating the stony pavement area (30 m deep) termed “Seaweed City”, dominated by red algae (Rhodophyta). Top left: *Wrightiella blodgettii*. Top right: *Hypoglossum hypoglossoides*. Middle right: *Gracilaria cylindrica*. Bottom left: *Jania capillacea*. Bottom right: *Tricleocarpa fragilis*.

**Figure 6:**
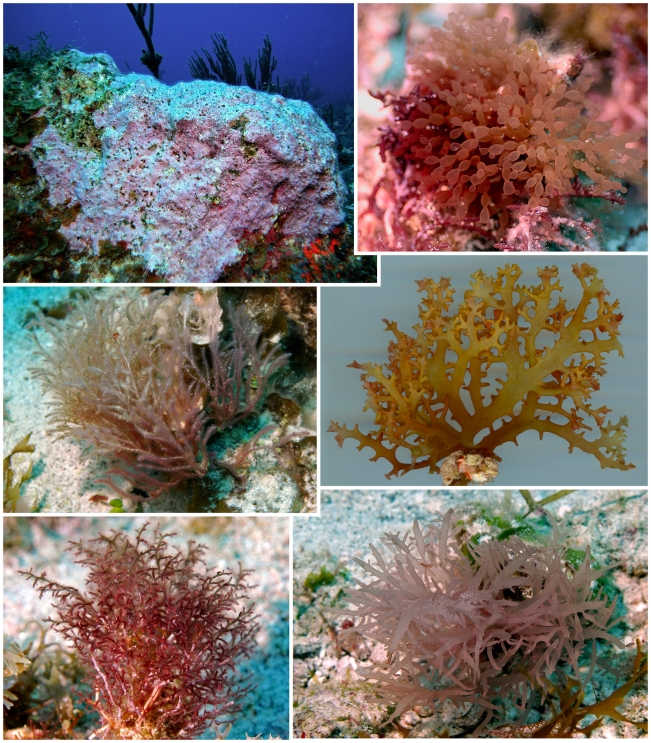
Selected specimens from “Seaweed City”. More Rhodophyta species characteristic of “Seaweed City” (Figure 5). Top left: *Porolithon pachydermum*. Top right: *Scinaia halliae*. Middle left: *Dasya antillarum*. Middle right: *Meristiella schramii*. Bottom left: *Laurencia intricate*. Bottom right: *Chrysymenia dickieana*.

**Figure 7:**
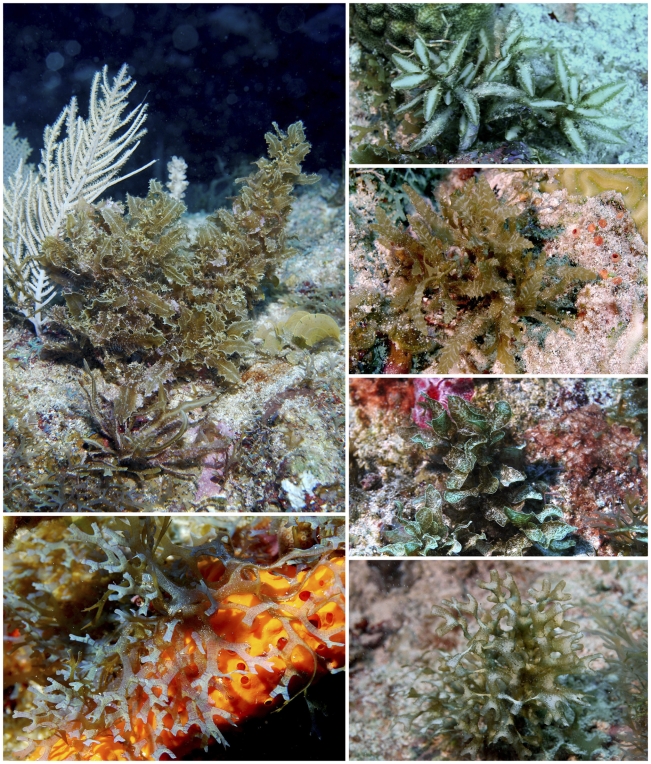
Selected specimens from potential new species. Some examples of potential new species of brown algae (Phaeophyceae) from Saba Bank (35–45 m deep). Top four: *Sargassum* spp. *Bottom* right and left: *Dictyota* spp.

Three sites surveyed were dominated by previously unknown unique algal communities. These included: (1) “Field of Greens” (N 17°30.620′, W 63°27.707′) characterized by an abundance by green seaweeds (Chlorophyta, Figure 3) as well as some filamentous reds, (2) “Brown Town” (N 17°28.027′, W 63°14.944′) dominated by large brown algae (Phaeophyceae, Figure 4), and (3) “Seaweed City” (N 17°26.485′, W 63°16.850′) with a diversity of spectacular fleshy red algae (Rhodophyta, Figures 5 and 6). Possibly 12 new species of brown algae (Figure 7) may be named following further scientific investigation. All of these macroalgae and their collection locations can be viewed as a virtual herbarium [11], where users can search for any combination of phylum, family, genus, species, infra-specific rank, author, collector, collector number and precise location as a satellite map and longitude/latitude. Searching for a given parameter generates information associated with the specimens collected by the project under that parameter. Images taken of in situ living plants from the field are attached at the bottom of the label data.

## Discussion

Prior to this survey, the two most diverse areas for algae reported in the Caribbean had been Diamond Rock, Martinique [10] and Pelican Cays, Belize [12], a mangrove, seagrass, and coral complex. Habitats on Saba Bank have far exceeded both of these places for species diversity per unit collection effort. A major reason for this uniqueness and richness is the sheer size and habitat range of the seamount/atoll.

**Figure 8:**
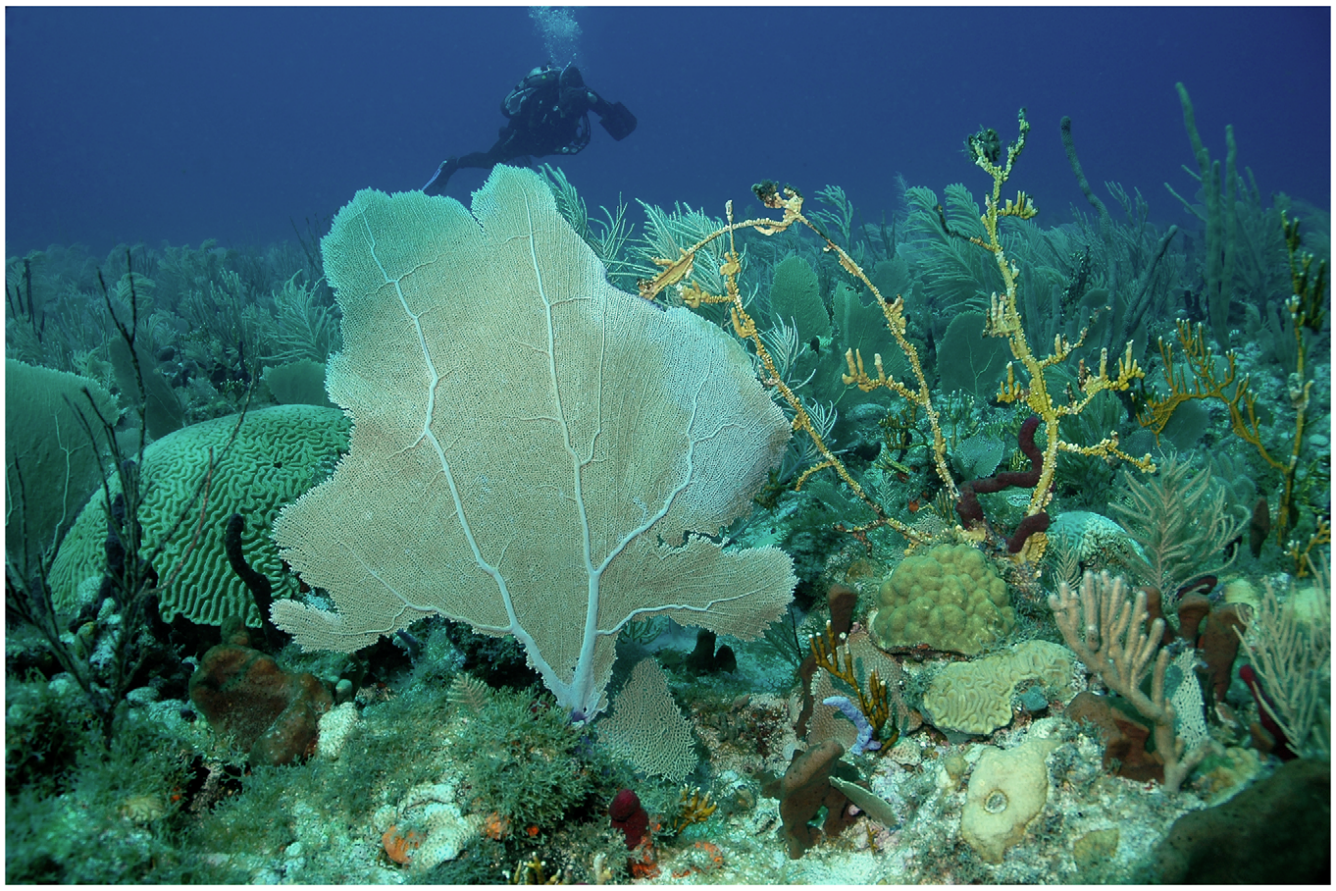
Windward fore-reef slope. Coral-dominated community characteristic of the windward (east) fore-reef slope of Saba Bank.

The rim habitats range from windward pristine coral reefs to extensive leeward rhodolith (coralline algal spheres) beds (N 17°25.832′, W 63°40. 962′) containing a high diversity of small epiphytic algal taxa. The Relative Dominance Model [9] is useful for characterizing the health of any given coral reef. According to this model, healthy coral reefs are dominated by reef-building (hermatypic) corals, crustose coralline algae, and high populations of herbivorous fishes – all characteristic of Saba Bank's windward eastern rim and fore-reef slope (Figure 8).

Vast sedimentary environments with some interspersed bedrock create relatively unstructured interior plains behind the rim communities, where seagrass beds would normally abound. However, seagrasses are absent (presumably due to excessive depths and insufficient light). Here vast plains of various groups of large and robust algal forms provide the three-dimensional structural heterogeneity. These are many of the rhizophytic (i.e., rooted) Bryopsidales forms typical of healthy seagrass beds in the sedimentary habitats. The seagrass model [13] indicates that these sand plains of robust large epiphyte-free green algae, as in Field of Greens (Figure 3), indicate pristine oligotrophic conditions. The same can be said in the cases of Brown Town (Figure 4) and Seaweed City (Figures 5 and 6), where especially clean robust macroalgal forms predominate on hard bottom. Few filamentous and thin sheet forms indicative of stressed or physically disturbed environments were observed.

## Materials and Methods

During 2-16 January 2006, we accompanied a multi-disciplinary team of sixteen other scientists and managers of marine protected areas to conduct a detailed biological assessment and sampling program of Saba Bank. The survey focused on the comprehensive survey of fishes, mollusks, crustaceans, macroalgae, and sponges. The team was supported by three vessels, two marine park patrol boats and a fishing vessel that operated together. Dives were made primarily in representative areas of interest at depths from 12 to 50 m and included both biological sampling and photographic documentation. A photographic overview of this expedition and the three dive sites with highest algal diversity is available online: http://www.littlersworks.net/.

Collections for pressing were taken by hand and preserved in the field and processed upon returning to the Smithsonian Institution. Nearly all specimens were photographed in situ and the same plants were placed in individual numbered plastic bags at the time of collection. These were transferred to polycarbonate scintillation vials, fixed in 5% formalin, finally preserved in 70% ethanol, and later examined microscopically. Additionally, identical specimens for molecular analyses were dried in frozen silica gel, placed in hermetically sealed vials, and stored in a freezer. All macroalgal/seaweed specimens were deposited in the Algal Collection of the US National Herbarium, Smithsonian Institution. All Saba Bank projects have collecting permits through CITES (where necessary) and the Saba Conservation Foundation (where CITES is not required).
